# Supplementary material for: Identification of the Extracellular Nuclease Influencing Soaking RNA Interference Efficiency in Bursaphelenchus xylophilus
Source: Int J Mol Sci. 2022 Oct 14;23(20):12278. doi: 10.3390/ijms232012278 (PMC9603779; doi:10.3390/ijms232012278)
Supplement: Supplementary file 1 [file ijms-23-12278-s001.zip › ijms-1959515-supplementary.pdf]

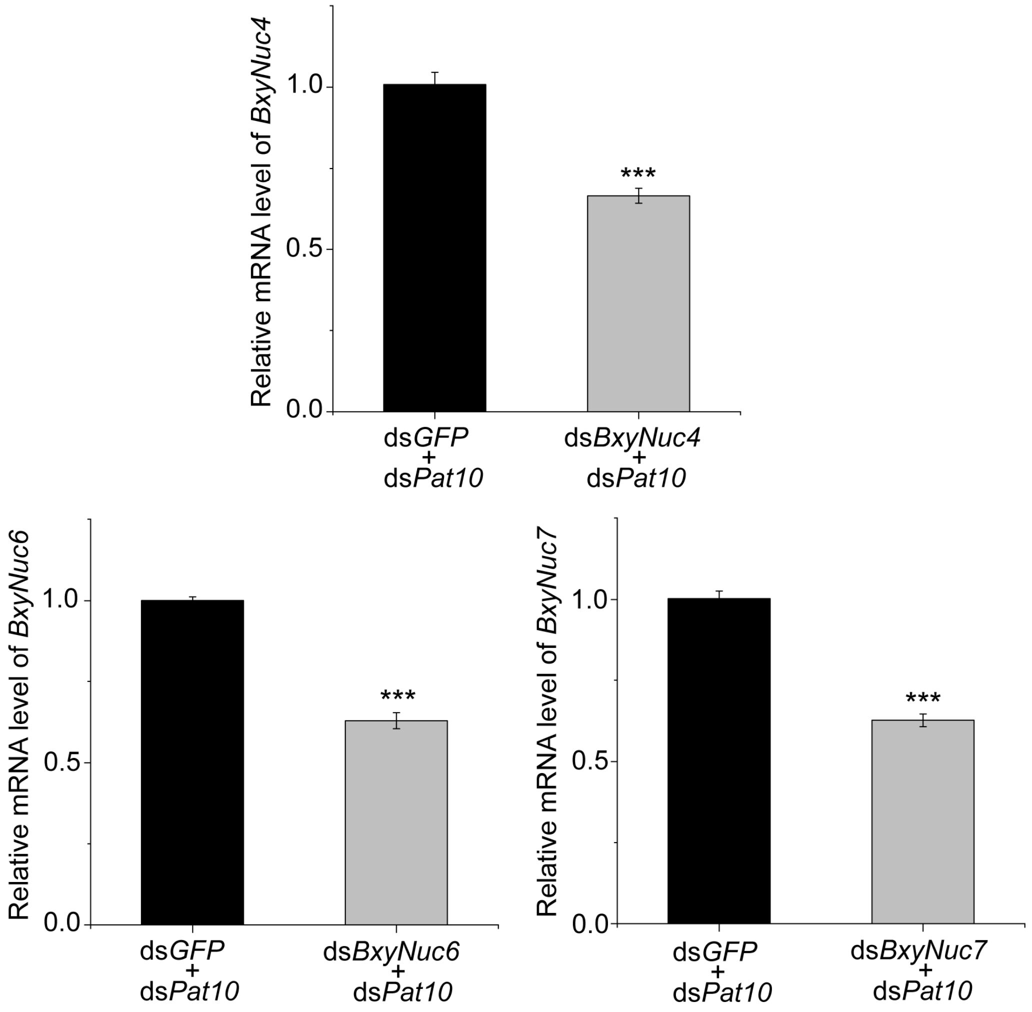

Figure S1: The relative expression level of *BxyNuc4, BxyNuc6 and BxyNuc7* after interfering with *Pat10* gene and nucleases *BxyNuc4*/*BxyNuc6*/*BxyNuc7* genes simultaneously for 24 h. Gene expression differences between two groups were calculated using the independent samples t test. Data are presented as mean ± SE, *** $P < 0.001$.;

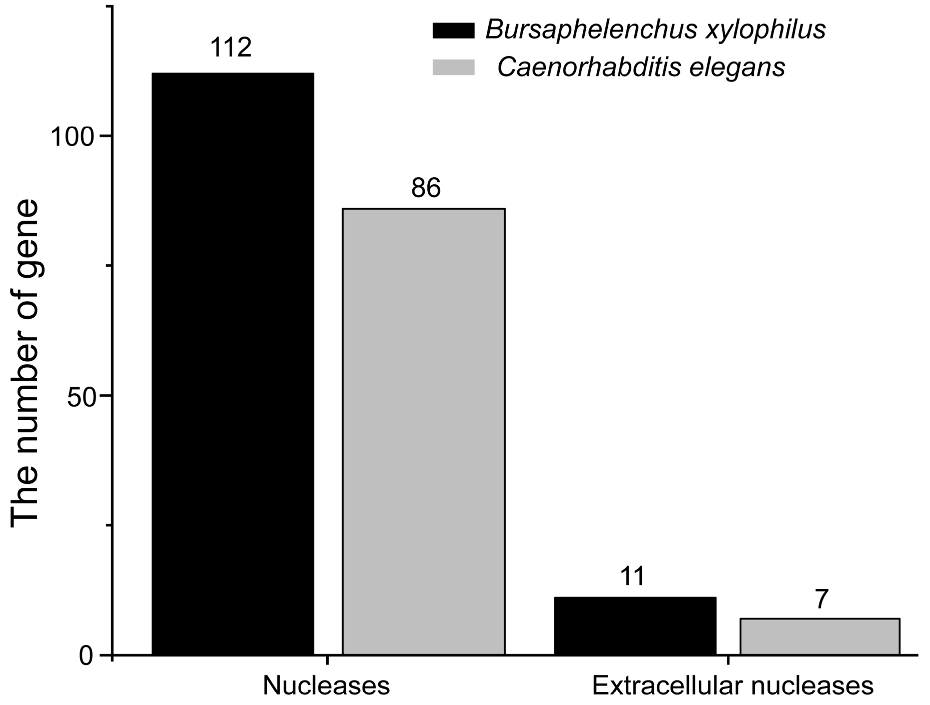

Figure S2: The number of nucleases and extracellular nucleases in *Bursaphelenchus xylophilus* and *Caenorhabditis elegans.*;

Table S1: List of primers;

Table S2: Information table of screened *B. xylophilus* nucleases;

Table S3: Comparison table between accession number and gene ID of cloned *B. xylophilus* extracellular nucleases.
